# Engaging South Asian Communities in the United Kingdom to Explore Infant Feeding Practices and Inform Intervention Development: Application of the REPLACE Approach

**DOI:** 10.1111/mcn.70009

**Published:** 2025-02-20

**Authors:** Kayleigh Kwah, Maxine Sharps, Naomi Bartle, Kubra Choudhry, Jacqueline Blissett, Katherine Brown

**Affiliations:** ^1^ Public Health and Applied Behaviour Change Laboratory University of Hertfordshire Hatfield UK; ^2^ Health and Life Sciences, School of Applied Social Sciences De Montfort University Leicester UK; ^3^ School of Social Sciences and Humanities Coventry University Coventry UK; ^4^ School of Life and Health Sciences Aston University Birmingham UK

**Keywords:** breast feeding, colostrum, culture, feeding behaviour, human milk, infant, social norms

## Abstract

Breastfeeding in UK Pakistani and Bangladeshi communities is positively and negatively influenced by cultural beliefs and practices. The LIFT (Learning about Infant Feeding Together) project aimed to understand the determinants of infant feeding in these target communities and to engage them in the development of a culturally specific and acceptable infant feeding intervention to support breastfeeding. Reported here is phase one of the LIFT project guided by the REPLACE approach (a framework for the development of community‐based interventions). The project involved an initial lengthy period of engagement with the target communities, using methods such as a community outreach event and identification of community peer group champions to help build trust. This was followed by iterative community workshops used to explore and build an understanding of infant feeding practices and the social norms and beliefs underlying these, and to assess community readiness to change. Consistent with previous research, the six key practices and beliefs identified from the workshops were: (1) Disparities between personal views versus cultural and normative barriers, (2) Family relationships and the influence on infant feeding decisions, (3) Pardah (modesty) and being unable to breastfeed in front of others, (4) Discarding colostrum (first breast milk), (5) Pre‐lacteal feeds (feeds within a few hours of birth and before any breast or formula milk has been given) and complementary feeding before the baby is 6 months old, and (6) The belief that bigger babies are better and that formula helps babies to grow. Participants perceived that Pakistani and Bangladeshi communities would be amenable to intervention that aimed to change some but not all of the infant feeding behaviours identified. Findings informed the co‐development of a culturally appropriate intervention toolkit to optimise infant feeding behaviour.

## Summary

- This is the first application of the REPLACE approach (a framework for the development of community‐based interventions) to the development of an intervention to increase breastfeeding.
- Culturally‐specific beliefs and behaviours (such as pre‐lacteal feeds and ‘pardah’) are having an influence on breastfeeding in UK Pakistani and Bangladeshi communities.
- Significant investment in community engagement is required to build trust so that culturally specific beliefs and behaviours such as these can be identified.
- Assessment of community readiness can be used to successfully identify beliefs and practices that are likely to be amenable to change and culturally acceptable.

## Graphical abstract

The LIFT project provides valuable insight into infant feeding practices of Bangladeshi and Pakistani communities in the United Kingdom. It identifies the communities' perspective on which infant feeding practices should be a priority and which are most amenable to change, offering a robust evidence base to draw on in the development of culturally appropriate intervention.

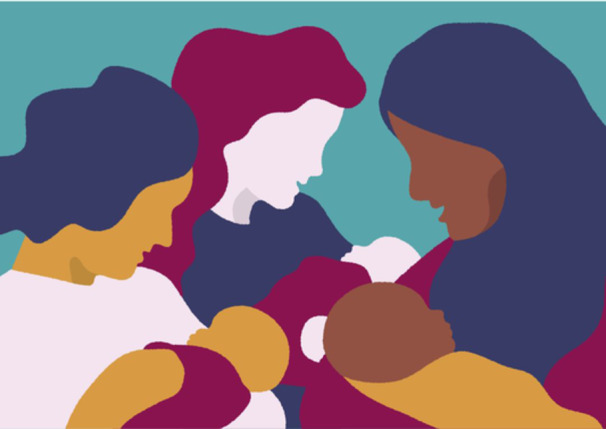

## 1 Introduction

The World Health Organisation recommends that all infants are exclusively breastfed for the first 6 months of life (World Health Organisation 2003), and the benefits of doing so are widely acknowledged and evidenced (Victora et al. 2016). However, across the world, and especially in the United Kingdom, statistics show that very few infants receive this (McAndrew et al. 2012). Furthermore, there is evidence of disproportionate rates of breastfeeding initiation across ethnic groups. Data from the UK‐based infant feeding survey, conducted last in 2010, indicated that, as a group, those of South Asian ethnicity had relatively high breastfeeding initiation rates (96%) compared to those of White British ethnicity (87%) (McAndrew et al. 2012). However, a re‐analysis of this data that separated the South Asian sub‐groups found that the high breastfeeding rates were driven by those of Indian ethnicity (97%), with lower proportions of Bangladeshi (85%) and Pakistani (79%) mothers initiating breastfeeding (Choudhry 2017). These rates indicate that babies born to mothers of Bangladeshi and Pakistani origin, living in the United Kingdom, may be vulnerable to lower breastfeeding rates than previously thought and potentially excluded from efforts to increase breastfeeding. The infant feeding survey was discontinued after the 2010 results, with national data subsequently reported by region but without reporting on data by ethnicity. The infant feeding survey has since been reinstated with data due to be released in 2025. Nonetheless, a more recent report that compares sources of data from NHS England and the Office for Health Improvement and Disparities (OHID) states that from 2020 to 2021, 69% of mothers from an Asian or Asian British ethnic group were breastfeeding either fully or partially at 6–8 weeks (Office for Health Improvement and disparities 2021).

Population‐level barriers to breastfeeding, as summarised in a recent UK review (National Guideline Alliance [UK] 2021), have been widely explored and cited in the literature. There is scant evidence, however, about how these might differ among the diverse UK population. While it has been demonstrated that some barriers are universal, such as perceptions of low milk supply, there is also evidence that others are culturally specific (Cook et al. 2021; Rayment et al. 2016). Even so, when barriers are similar, the underlying beliefs can be different, and the social environment can exert different types of influence (Choudhry and Wallace 2012; Cook et al. 2021; Rayment et al. 2016; Twamley et al. 2011).

Despite breastfeeding being recommended and supported by religion (Islam), some social and cultural expectations within Bangladeshi and Pakistani families in the United Kingdom have been shown to affect infant feeding practices (Choudhry 2017; Choudhry and Wallace 2012; Manikam, Lingam, et al. 2018; Rayment et al. 2016; Twamley et al. 2011b). These include pre‐lacteal feeds (Choudhry 2017), early introduction of complementary feeding (Choudhry 2017; Twamley et al. 2011), discarding colostrum or delaying the introduction of breast milk (Choudhry 2017) and ‘Pardah’ that prevents breastfeeding in the company of others (Choudhry and Wallace 2012).

In Pakistani and Bangladeshi communities, it is customary to pass down information from older to younger members of the family. Evidence has identified that some Pakistani grandmothers believe that colostrum (first breastmilk often described as being golden in colour) should be discarded in favour of ‘fresher’ breastmilk (Ingram et al. 2003), and also that some Bangladeshi and Pakistani grandmothers believe that there is a need for babies to be offered (boiled) water alongside breastmilk (Ingram et al. 2003). While the younger generation has been identified as less likely to express these beliefs or cite them as ‘outdated’ (Rayment et al. 2016; Twamley et al. 2011), healthcare workers remain concerned about these practices (Twamley et al. 2011), and it is possible therefore that they continue to have an impact on breastfeeding in this population. Although evidence indicates that the older generations in Bangladeshi and Pakistani families know the benefits of breastfeeding and support its initiation (Ingram et al. 2003a), there is also evidence that they frequently encourage the use of formula milk, especially to help babies through their first few days (Rayment et al. 2016), a practice which can negatively impact the establishment of breastmilk supply. Often advice given by family members about complementary feeding practices is followed due to a sense of obligation to community elders, despite it conflicting with advice from health professionals (Manikam, Lingam, et al. 2018). It is also known that expectations about family roles and responsibilities in such communities have led some mothers to stop exclusively breastfeeding (Choudhry and Wallace 2012; Ingram et al. 2008; Rayment et al. 2016; Twamley et al. 2011b). This body of evidence clearly demonstrates that decisions to initiate and continue breastfeeding occur within a wider socio‐cultural context, with the role of others being critical to the outcome.

The reasons surrounding the decision to breastfeed and the performance of cultural practices that may impact this differ by community and can be driven by different belief systems and social norms. It is imperative to recognise and understand this diversity when developing interventions. The UK Medical Research Council (MRC) who funds research aiming to improve human health and works closely with the NHS and health departments, published guidance on the development of complex interventions. This guidance identifies high‐quality formative research as essential (Skivington et al. 2021), with emphasis placed on including all who have a stake in the project. This is important for many reasons, but particularly to understand the socio‐cultural and context‐specific determinants that influence the beliefs and behaviours of specific populations. Significant investment in community engagement, particularly when there are cultural sensitivities, can enrich this understanding and build trust between members of the community and other stakeholders, including academics. Together, increased trust and understanding results in interventions tailored to local circumstances that are most likely to work (Craig et al. 2008). The LIFT project (Learning about Infant Feeding Together), outlined in this paper, aimed to understand the determinants of infant feeding in Pakistani and Bangladeshi communities by engaging them in the development of a culturally specific and acceptable infant feeding intervention to support breastfeeding.

Research has shown that although UK maternity and breastfeeding services have introduced efforts to increase the cultural competency of these services, achieving this to a high and consistent standard requires further investment (Hassan et al. 2020; McFadden et al. 2013; Wilde 2023). This highlights a need for tailored interventions designed to address the specific needs and socio‐context of this population. Such interventions targeting infant feeding‐related behaviours in the first 6 months of an infant's life in the United Kingdom are lacking. However, one noteworthy intervention, developed by Ingram and colleagues (Ingram et al. 2003; Ingram and Johnson 2004), was delivered to South Asian mother–grandmother or mother–partner pairs and had a positive impact on infant feeding behaviours (Ingram et al. 2003). It was seen to be acceptable, enjoyable and useful by the communities (Ingram and Johnson 2004). However, this intervention, is now over 20 years old, with no further development or testing published. There is, therefore, a need for a theoretically informed, culturally tailored intervention to support early feeding in UK South Asian communities. Reported here is phase one of the LIFT project guided by the REPLACE approach.

The REPLACE approach (Barrett et al. 2015) is a framework for the development of community‐based interventions which recommends the application of both individual and community‐level behaviour change theories to support and inform the co‐production of interventions. Exploring the belief systems in partnership with community members and working collaboratively to co‐create intervention messages and materials is fundamental to the approach. The first three steps of this approach, referred to as ‘elements’, propose active community participation with the aim of: developing trust and a commitment to the project, understanding social and cultural norms and beliefs impacting on the target behaviour, and ascertaining the community's readiness to engage in efforts that encourage change. The REPLACE approach has been used with communities in the United Kingdom and Europe to explore and develop interventions to address the cultural practice of FGM (female genital mutilation) and research has suggested that the application of this approach has the potential, over time, to bring about changes in practice norms and attitudes (Barrett et al. 2020). Furthermore, the REPLACE approach aligns strongly with the MRC guidance for the development of complex interventions, specifically the importance of community engagement and the value of exploring socio‐cultural determinants of behaviour and developing interventions together with these communities.

Phase one of the LIFT project used the first three elements of the REPLACE approach to develop a culturally specific intervention to increase breastfeeding among Bangladeshi and Pakistani communities. This paper outlines how the approach was used to engage the community, understand social and cultural norms and beliefs around breastfeeding, and assess community readiness to change. Each of these steps is considered a prerequisite for determining the content of culturally competent behaviour change interventions. The paper offers insights into the challenges experienced as well as the benefits of utilising this approach for the target community that could help to inform others undertaking similar work with communities. It outlines and explicitly maps the findings of this formative research to an evidence‐based framework (The REPLACE approach). The findings of this process were then used to inform the co‐development of an evidence‐based, culturally sensitive intervention to address behaviours the community felt they were most ready to change. This fourth element of the REPLACE approach ‘intervention development’, is reported elsewhere (see Kwah et al. forthcoming).

## 2 Methods

### 2.1 The LIFT Project

The LIFT project aimed to develop a novel, culturally specific and acceptable intervention to promote optimal infant feeding practices among Pakistani and Bangladeshi families living in the United Kingdom. Engaging with and building trust with the local community was central to the project and its approach to (a) understand the determinants of infant feeding behaviours, (b) understand the potential for change around infant feeding behaviours and (c) develop resources that the community can use themselves to optimise infant feeding. The project was guided by the principles of the REPLACE approach (see below) and conducted by a multi‐disciplinary team. The project was based in a UK city in the Midlands (Coventry) where the second largest broad ethnic group is Asian or Asian British (18.5%) (Office for National Statistics 2021).

### 2.2 The LIFT Project Team

The LIFT project team involved a close partnership between academics and a third‐sector organisation, Foleshill Women's Training (FWT) with expertise related to infant feeding. FWT offers culturally sensitive support to women from diverse communities in the UK Midlands (particularly those from ethnic minority groups), assisting access to education, healthcare and employment. They offer a specific programme around the perinatal period, which includes infant feeding support (established existing service). The authors of this paper, comprising the academics on the team (referred to as the LIFT research team), had prior experience in infant feeding‐related research and expertise in the field and have lived experience of breastfeeding, including problems with breastfeeding. One of these authors also identifies as belonging to the ethnic background of one of the target communities.

### 2.3 The REPLACE Approach

**Figure 1:**
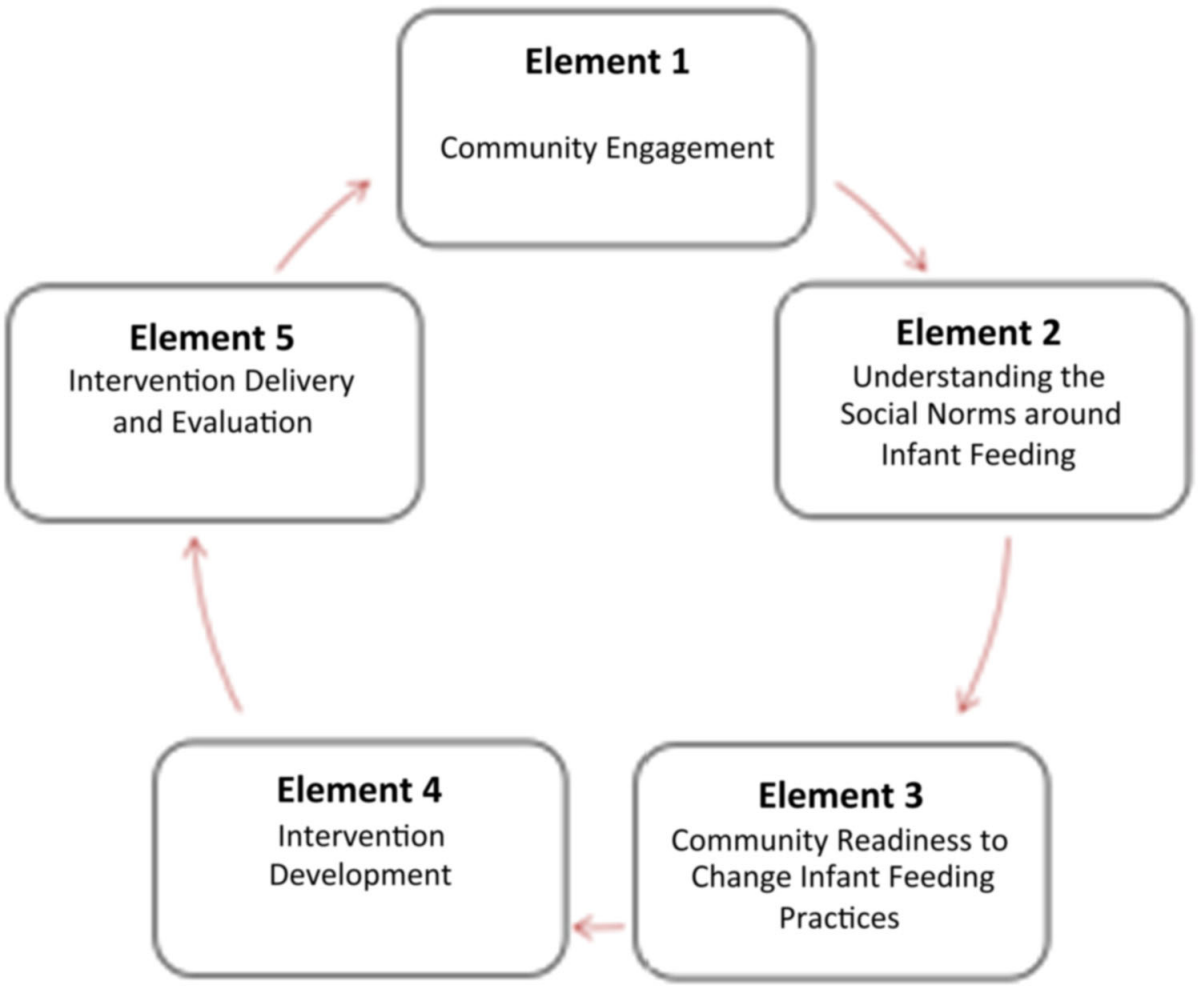
An adapted REPLACE cyclic framework for social norm transformation for infant feeding.

The REPLACE approach (Barrett et al. 2015; Barrett et al. 2020; Brown et al. 2013) involves five elements in total. See Figure 1 for an adapted REPLACE cyclic framework from Barrett et al. (2015).

#### 2.3.1 Element 1–Community Engagement

The main objectives of element 1 are to identify the target community and key stakeholders, appoint community‐based researchers and/or community peer group champions, and gain the trust of the target community. To meet these objectives, the LIFT project team undertook the following activities:

- A community family event to introduce the project, and build familiarity and trust between the community and the team. A local community centre was chosen for this event as it was familiar and accessible to the families in the community. The event was advertised by FWT throughout the community using posters, fliers, their website, social media feeds, and word‐of‐mouth across their networks. While the event was primarily hosted for this purpose, a range of stalls and activities for families (such as a bouncy castle, henna hand/body art, food stalls, and free refreshments) were put on to encourage attendance and set the tone for a community partnership. One stall provided information about the LIFT project, and an opportunity to chat to research staff about getting involved, and leave contact details. Those who signed up were formally invited to join a series of community workshops (see element 2).
- Identification by FWT of key stakeholders in the community who may (directly or indirectly) have an affect on, or be affected by, infant feeding‐related behaviours. This was done by considering a number of questions outlined in the REPLACE approach (Whose well‐being is affected?; Who are supporting social norms related to infant feeding decisions?; Who is enforcing social norms related to infant feeding decisions?; Who has the knowledge and skill to bring to intervention development?; Who are the influential groups?; Who are likely to contribute to the delivery of the intervention?
- Recruitment of community peer group champions (whose role was to encourage and assist other women in the community to participate in the project and co‐develop an infant feeding intervention) using the following three methods:

1. Poster advertisements were placed in the FWT centre, and the FWT team identified and made direct contact with prominent female members of the Bangladeshi and Pakistani communities.
2. At the above community family event, women were encouraged to talk to the LIFT project team and could express interest in becoming a community peer group champion.
3. During the community workshops (see element 2), women were encouraged to talk to the LIFT project team and could express interest in these roles.

#### 2.3.2 Element 2–Understand the Beliefs and Practices Surrounding Infant Feeding

Element 2 of the REPLACE approach aims to understand the belief systems and social norms in the community and identify any barriers to changing these. This incorporates information collection, analysis and interpretation, and was primarily achieved via a series of community workshops. Activities here also served to continue the process of community engagement and building trusting relationships between community members and the LIFT project team.

A series of four community workshops were planned and held in a familiar community space, attended by women identified via the methods described in element 1 above. The workshops were structured in a way that gave people the freedom to engage at a level they were comfortable with, or indeed to leave on their own terms if they so wished (natural breaks in the timetable were provided to allow for this). The workshops were held from 9.30 AM–12.30 PM to accommodate time for school drop off. Children were welcome and travel expenses were provided. Refreshments were provided. The planned structure and content of the workshops are outlined in Table 1 below.

**Table 1:** Details of community workshops.

Workshop topic	Workshop content	Structure
Personal breastfeeding journeys	Discussions were held about personal breastfeeding experiences or journeys. There was an option to create posters using pre‐supplied images.	Two workshops were held and participants were invited to attend one of these workshops.
Pros and cons of breast and formula feeding	Posters about the pros and cons of breastfeeding and bottle feeding were developed. A researcher facilitated open discussion around the table and added content to a group poster. Participants were encouraged throughout to add ideas and thoughts using post it notes, and other researchers added any new thoughts that arose from break out conversations.	Two workshops were held that had the same focus. One workshop was held with each of the community groups (Pakistani and Bangladeshi).
How to optimise infant feeding in the community	Discussions were held to identify areas where attendees would like to see change. Pre‐written scenarios were used to elicit suggestions for how to improve infant feeding practices.	Two workshops were held that had the same focus. One workshop was held with each of the community groups (Pakistani and Bangladeshi).
Thank you event and collective celebration event for Eid	The key findings from the community workshops were summarised and presented back to the participants for further discussion. The event also celebrated the end of the project and the festival of Eid.	One event was held for all the members of the community

Initially, each workshop was planned as a focus group discussion and relevant topic guides and materials were developed accordingly. It quickly became apparent, however, that these methods were both impractical and inappropriate for the group and the setting. As a result, an adapted approach was taken to all of the workshops, which was more creative, pragmatic and flexible. This involved a combination of group discussion, smaller break out group discussion, and storytelling. Flipchart paper and Post‐it notes were provided, and participants were invited to write about their experiences. In most cases, participants preferred to talk, and members of the LIFT project team made notes on their behalf. Members of the LIFT project team also acted as interpreters for some participants allowing them to speak the language of their choice (Bengali, Urdu and Punjabi). No recordings or transcriptions of the discussions were made. Instead, data from the workshops consisted of hand‐written notes capturing the thoughts and views of attendees; this was supplemented by notes made by the team recording aspects of the process and early reflections. The LIFT research team conducted a rapid content analysis, grouping data into semantic themes.

#### 2.3.3 Element 3–Community Readiness to Support Breastfeeding

Element 3 of the REPLACE approach involves assessing the community's readiness to change (Plested et al. 2006). The main objective is to match the community stage of readiness with the intervention goals to ensure it is appropriate and likely to be effective. Though the REPLACE approach recommends recruiting a range of community members and conducting interviews to determine this, an adapted approach was taken because of the relatively tight funding timeframe and because community workshops had already provided insights into the beliefs and cultural norms influencing infant feeding behaviours. The LIFT team, with the support of some of the community members, discussed the community's readiness to change infant feeding‐related beliefs and practices that were identified from the community workshops. Readiness to change was classified as low, medium or high, using a nine‐point scale as a guide (see Table 2).

**Table 2:** Stages of community readiness to change infant feeding behaviours.

Low (1–3)			Medium (4–6)			High (7–9)		
1–No community awareness (of the issue)	2–Community denial/resistance	3–Vague community awareness	4–Preplanning	5–Preparation	6–Initiation	7–Stabilisation	8–Expansion	9–Community Ownership

#### 2.3.4 Element 4–Intervention Development

Element 4 of the REPLACE approach, which involves the development of the intervention, is beyond the scope of this paper. Methods used in this stage are reported elsewhere (Kwah et al. forthcoming).

#### 2.3.5 Element 5–Intervention Delivery and Evaluation

Element 5 of the REPLACE approach, which involves the delivery and evaluation of the intervention has not yet been completed. Future work will be conducted and reported in due course.

### 2.4 Ethical Statement

Ethical approval was received from Coventry University's ethics committee.

## 3 Results

### 3.1 Element 1–Community Engagement

The process of community engagement spanned 8 months from the first family event to the last community celebration workshop. The LIFT project team members from FWT brought a significant amount of knowledge of the community, enabling the team to have a good level of understanding about the community before embarking on the project, and their established relationships aided community mobilisation. This was particularly important in the community engagement element of the project, as researchers can be deemed as ‘cultural outsiders’.

#### 3.1.1 Identifying the Target Community

Determining and choosing the target community was predefined by the project's aims as: Pakistani and Bangladeshi communities living in Coventry (a city within the West Midlands region of England, UK).

#### 3.1.2 Community Family Event

The community family event was held in November 2017 and attended by approximately 200 local community members. Members of the LIFT project team spent the day interacting informally with community members, introducing them to information about the LIFT project, who the team were, what the project was aiming to achieve, and how, and inviting people who were interested to sign up to find out more. Approximately 30 community members expressed an interest in the LIFT project on the day.

#### 3.1.3 Stakeholders

FWT identified the following stakeholders as affecting or being affected by infant feeding‐related behaviours (Table 3).

**Table 3:** Key stakeholders in infant feeding related behaviours.

People affected Expectant mothers and fathers New mothers and father Infants
Influential people Grandparents/elder community members Health professionals Community leader Trusted local support services
Service providers Health professionals (midwives and health visitors) Locally run organisations/grassroots 3rd sector providers of support for families

#### 3.1.4 Community Peer Group Champions

Six women were recruited as community peer group champions via the various methods described. All were women, were from the Pakistani and Bangladeshi communities in Coventry and were mothers. All participated in the community workshops and went on to co‐develop the intervention in partnership with the LIFT project team.

#### 3.1.5 Gain Trust and Buy‐In of the Target Community

The number of attendees at the community event indicated that the LIFT project had some initial buy‐in from the community and that the community was willing to engage in the project. The recruitment of six community peer group champions was also promising, indicating that women were interested in the issues, and willing to work with the LIFT project team. Buy‐in was later built on through the community workshops.

#### 3.1.6 Challenges to Engaging the Community

Challenges encountered included the attendees not always being on time and sometimes wanting to discuss topics unrelated to infant feeding. Traditional focus group approaches were not appropriate as they were deemed too formal, and there were often different languages being spoken. Some women had difficulties with written tasks and were hesitant to engage in utilising creative resources that had been designed to offer alternative ways to describe their experiences and share their thoughts. Therefore, the methodological approach taken included more flexible and pragmatic approaches (see Section 2).

### 3.2 Element 2–Understand the Beliefs and Practices Surrounding Infant Feeding

The workshops were run between November 2017 and June 2018. See Table 4 for details.

**Table 4:** Community workshop attendance.

Workshop	Number of attendees
Community workshop 1	16
Community workshop 2 (conducted twice, one for each community group)	29
Community workshops 3 (conducted twice, one for each community group)	18
Community thank you event and collective celebration event for Eid	51

The first community workshops were held with a mix of mothers and grandmothers from both Pakistani and Bangladeshi communities, however, subtle differences arose (e.g., views and beliefs about colostrum), and thus all further workshops were run separately for each community group to allow for free exploration of specific beliefs in a safe and supportive environment. The final community thank you event, which included celebrations for Eid, was attended by all the community members.

The themes generated were rooted in narratives about social and cultural norms. Six themes were identified and are summarised below.

#### 3.2.1 Theme 1: Personal Views Versus Cultural and Normative Barriers

The participants recognised the benefits of breastfeeding, including that breastfeeding provides nutrients, helps the baby to grow and is a way of building the baby's immunity. Most participants spoke (retrospectively) about their desire and intentions to breastfeed their child, however, they went on to discuss a number of cultural and normative beliefs that acted as barriers, which conflicted with these personal views. These impacted their breastfeeding journey and are presented below.

#### 3.2.2 Theme 2: Family Relationships and the Influence on Infant Feeding Decisions

##### 3.2.2.1 Sub Theme 2.1: The Role of the Mother‐in‐Law

Family members were described as being important in infant feeding decisions. A woman's mother‐in‐law, in particular, was frequently cited as having a key role and important influence on infant feeding and, for some, was experienced as a barrier to breastfeeding. Within Bangladeshi and Pakistani cultures, the woman traditionally lives with her husband and his family, and the mother‐in‐law is typically considered the head of the house. The participants reported that typically mothers‐in‐law did not have much knowledge about breastfeeding and often requested that the baby be given formula as they believed that it fulfilled the baby's nutritional needs better than breast milk. The participants also said that they personally felt an obligation to do as the family elders asked and did not feel comfortable having conversations with their in‐laws about how they wished to feed their baby.

##### 3.2.2.2 Subtheme 2.2: The Role of the Infant's Father

The participants also described how the fathers of the infants did not feel that feeding the baby was their business, and they did not offer any advice regarding infant feeding. However, they suggested that including the father in infant feeding decisions would be a positive step and that they would like the father to be more supportive and involved in infant feeding decisions and support. Furthermore, they suggested that fathers could and should play an important role in supporting mothers to breastfeed by speaking to their own mothers (the mother‐in‐law) about infant feeding decisions and intentions. The participants felt that having the fathers involved in this way would help mothers feel more comfortable expressing their views to the wider family.

##### 3.2.2.3 Subtheme 2.3: Dual Role as a Daughter‐in‐Law and Mother

The participants described how women are viewed to have two roles; one as a mother and one as a daughter‐in‐law. They explained that their role as a daughter‐in‐law involved responsibilities such as cooking, cleaning and caring for elders in the family and that breastfeeding was perceived to be difficult due to these responsibilities. One of the participants shared that their mother‐in‐law thought that the baby should only be attended to by the mother once the chores were finished, and several of the participants talked about how formula feeding meant that the baby could be fed by other people, enabling mothers to fulfil other responsibilities.

#### 3.2.3 Theme 3: Pardah (Modesty)

Pardah, meaning ‘screen’ or ‘veil’, was discussed as a barrier to breastfeeding, both in and outside the family home. The term is used in Muslim and Hindu communities to refer to both a cultural and religious social practice of female seclusion and typically involves a requirement to cover the skin and the female form, generally, particularly in the presence of men. The participants spoke about how they had felt they could not breastfeed in front of their family members and how they would need to go to another room to do so. For this reason, they talked about opting to formula feed as a direct result of feeling unable to breastfeed in the presence of others.

#### 3.2.4 Theme 4: Colostrum

The participant's discussions about discarding colostrum were mixed. In general, the Bangladeshi participants said that their community discarded colostrum because it is perceived to be ‘old’ or ‘dirty’, whereas the Pakistani participants discussed how they believed colostrum was good and did feed this to their babies.

#### 3.2.5 Theme 5: Pre‐Lacteal Feeds and Complementary Feeding

The participants discussed pre‐lacteal feeds as being common when they had their babies and talked about how a family elder gave their babies honey or dates (chewed up) when they were newborn, before the baby was fed any milk for the first time. They described how this practice, known as ‘ghutti’, is believed to pass on important qualities from the elder to the baby, and that it is viewed as an important part of their religion and culture.

Early complementary feeding was discussed as being common, with babies being offered juice or water and soft foods. The participants talked about the cultural tradition to offer water mixes (e.g., water mixed with herbs) that are viewed as beneficial and that they were encouraged to give these to their babies. For example, lychee and cardamom were believed to prevent stomach problems; boiling water mixed with dill seed was believed to help clean the insides after the baby was born; honey and cardamom seeds were used to help the baby sleep and were believed to make the baby stronger; and saffron was believed to help ease wind.

#### 3.2.6 Theme 6: Baby's Size

The participants talked about beliefs within these communities that a bigger baby was better and that the size of the baby was linked to the quality of the breastmilk. There was a view that some mothers' milk was better than others, and if the baby was strong and healthy, then this was attributed to their mother's milk. The benefits of the mother's milk were sometimes referred to as ‘Maa Ka Dood’ (the importance of mother milk), which has also been found in previous research, where women used this term to describe psychological benefits (Choudhry and Wallace 2012). However, if a baby is small, then it is believed that the mother's milk is not good enough, and formula or food should supplement or replace breastmilk. The participants talked about the fact that some in‐laws (mostly the mother‐in‐law) believe that formula milk feeding ensures that the baby gets enough milk.

### 3.3 Element 3: Community Readiness to Change

#### 3.3.1 Identifying Which Beliefs and Practices to Target

A community readiness to change discussion concluded that most of the beliefs and practices were open to change (see Table 5).

**Table 5:** Readiness to change infant feeding‐related beliefs and practices identified from community workshops.

Belief/practice	‘Readiness to change’ narrative	Stage of change
**Family relationships and the influence on infant feeding decisions** Breastfeeding decisions and behaviour are influenced by family members (e.g., mothers‐in‐law make decisions, and fathers of babies do not have a role in infant feeding behaviour). Daughters‐in‐law are responsible for the household; breastfeeding is perceived to be difficult due to these responsibilities. ‘Pardah’ or the need for modesty, making bottle feeding easier and breastfeeding difficult in the presence of others.	Value is placed on breastfeeding at all levels of the community and is seen to be supported in religion. However, infant feeding decisions are not always solely made by the parents of the baby, and often older members of the family (seen as head of the household) request that the baby is fed formula, for a number of reasons. This is a decision that new parents may not feel confident to argue and new fathers do not tend to view that they have a role in the decision making about infant feeding choices. Community peer group champions believed the community was ready to start addressing this.	Medium
**Colostrum (first breast milk) is ‘old’ or ‘dirty’ and is discarded before the infant's first feed** [Bangladeshi community]	The women felt that if the beliefs about old or dirty milk are present, this is likely to have come from elders. Thus, messaging needs to be meaningful to them and/or support mothers to discuss with their elders. Community peer group champions believed the community was ready to start addressing this.	Medium
**Pre‐lacteal feed or ‘Ghutti’ passes on important qualities from family elder to baby**	Pre‐lacteal feeds hold significant meaning to families and are not considered to be harmful. It was believed that it was just a tiny taste and did no harm, and the process of the elder passing on their qualities to the baby was seen as an important cultural practice. Although pre‐lacteal feeds are not in the Qu'ran (central religious text of Islam) they are described in the Hadith (oral tradition) regarding what the Prophets did. Families that follow these teachings are unlikely to be influenced. The documents indicate that honey or chewed‐up dates can be given, so alternatives are unlikely to be accepted. The LIFT research team members believed that the intervention toolkit should aim to stop pre‐lacteal feeds due to the potentially negative health consequences of this practice. However, representatives of the third‐sector organisation (FWT) believed that pre‐lacteal feeds were a sensitive cultural practice, and targeting this may lead to the alienation of some of the women and disengagement with the toolkit. The Community peer group champions also believed that pre‐lacteal feeds were an important cultural practice which should not be stopped, however, they agreed that changing the type of food offered as a pre‐lacteal feed was possible, and messages around avoiding honey (due to the risk of Botulism) were potentially acceptable. Therefore, it was decided as a group that pre‐lacteal feeds could be included in an overarching message focussed on the avoidance of complementary feeding before the baby is 6 months old.	Low
**Giving ‘tastes’ of other foods/drinks (aside from breast or infant formula milk) before 6 months of age**	The community tends not to see this as giving food but as giving ‘tastes’ which make it difficult to address. This is considered important, to expose baby to tastes early. Community peer group champions believed that members of their community would be open to the idea of preventing complementary feeding in the first 6 months of their baby's life.	**Medium**
**Some mother's milk is not good enough quality and results in a smaller, less nourished baby, who needs to be given formula milk** The belief that formula milk = bigger and healthier baby (mostly from the elder generation)	Value is placed on breastfeeding at all levels of the community and supported in religion. Community peer group champions believed the community is ready to address barriers to enable mothers to breastfeed as they seem to wish to.	**Medium**

Intervention development (element 4), drawing on the beliefs and practices identified (element 2), along with the assessment of whether the community was ready to change (element 3), was the final stage of the REPLACE process and is reported elsewhere (see Kwah et al. forthcoming).

## 4 Discussion

The findings collated from the LIFT community workshops add to the body of research by providing further insight into the social and cultural beliefs and practices surrounding breastfeeding in Bangladeshi and Pakistani families in the United Kingdom. Gathered through a period of engagement (8 months) with the community and a partnership with a local trusted organisation, the main findings suggest that Pakistani and Bangladeshi women express a desire and intention to breastfeed, can identify the benefits for baby, and understand the cultural and religious importance of breastfeeding, as has been reported in previous research (Choudhry 2017; Choudhry and Wallace 2012; Ingram et al. 2008; Rayment et al. 2016; Twamley et al. 2011). However, a number of culturally specific practices present challenges with establishing breastfeeding and often were heavily influenced by the older generation's recommendations about infant feeding decisions for their baby. As described in other research with South Asian populations, the mother‐in‐law (or other family members) often plays an important role in influencing the decisions made about an infant's feeding, with advice often conflicting with that given by health professionals (Manikam, Prasad, et al. 2018). Although the participants in this study did not feel their family members were necessarily knowledgeable about breastfeeding, they felt unable to discuss other views that went against their advice, even when it conflicted with advice from health professionals. Furthermore, as evidenced previously (Ingram et al. 2008), they felt that the fathers did not perceive they had a role in infant feeding decisions and similarly would not discuss it with the family. Beliefs interfering with breastfeeding were commonly expressed, such as the encouragement to use formula or introduce solids earlier to meet perceived unmet nutritional needs and to create bigger babies viewed as healthier (Lakhanpaul et al. 2020). Cultural practices were also identified as influencing infant feeding practices. ‘Pardah’, meant that the women did not always feel able to breastfeed in or outside the home, a finding that has been reported elsewhere (Choudhry and Wallace 2012; Ingram et al. 2008). Furthermore, pre‐lacteal feeding and complementary feeding before 6 months were discussed as being common practices among both communities, but consistent with other research, there are differences in beliefs about discarding colostrum (Choudhry 2017; Ingram et al. 2003). Furthermore, breastfeeding was described as making the woman's role as a daughter‐in‐law (which included cooking, cleaning and caring for elderly relatives) more difficult, and formula feeding meant other family members could feed the baby while the woman fulfilled this duty, again consistent with other research (Choudhry and Wallace 2012; Ingram et al. 2008; Rayment et al. 2016; Twamley et al. 2011).

These findings contribute to the limited body of existing evidence on culturally specific beliefs about breastfeeding. Specifically, they offer insight into the beliefs held by two UK ethnic groups, namely Bangladeshi and Pakistani communities, which will inform the development of a culturally specific intervention. This research adds to our understanding by also providing insight into which behaviours the communities would like to see a change in and how they assess the community's readiness for that change. In other research, although beliefs about practices such as discarding colostrum have been identified, it has been reported that younger generations view this as outdated or no longer being carried out (Rayment et al. 2016; Twamley et al. 2011). The present study, however, clearly demonstrates the ongoing influence of elders and the challenge of discussing infant feeding. Nonetheless, this research indicates that the UK Pakistani and Bangladeshi communities would be amenable to an intervention aimed at changing infant feeding‐related behaviour. For example, participants expressed wanting men to be more involved in infant feeding decisions and support and suggested that they could offer support for breastfeeding by having conversations with family members about how new parents would like to feed their baby. Additionally, although they felt pre‐lacteal feeds are important within their community and represent the passing of important qualities from an elder to an infant, they also recognised the health concerns, especially the use of honey. The assessment of the community's readiness to address this and their insights into the belief resulted in important discussions and suggestions about how to sensitively address this behaviour. Without the team's careful approach to exploring and understanding this issue, the importance of how to address this practice may have been missed, ultimately resulting in intervention efforts being inappropriate and ill‐informed. The process of intervention development and how this research was used to inform the co‐design of a culturally appropriate toolkit of resources is reported elsewhere (Kwah et al. forthcoming).

The role of an existing and embedded community organisation in gaining the trust of the community and recruiting community peer group champions was essential and core to the success of the project. Without the third‐sector organisation partnership, community engagement would not have been achieved as rapidly or as successfully. The original aim was to recruit one male and people representing all age groups with different levels of influence in the community, however recruitment of men was unsuccessful and on reflection would have resulted in the need for additional gender‐specific workshops due to social difficulties perceived in discussing breastfeeding. Further work to understand the perspectives of other members of the Pakistani and Bangladeshi communities is warranted, especially men, because the women involved were keen for them to take a more active role in infant feeding decisions. A further consideration that would have added value to the understanding of the community's beliefs would have been to explore the impact of acculturation to breastfeeding and other infant‐related behaviours. Previous research has shown that acculturation may affect South Asian women's breastfeeding intention and behaviours (Choudhry and Wallace 2012) and, therefore, may have impacts on the design and delivery of intervention efforts.

The LIFT project provides the first application of the REPLACE approach principles (Barrett et al. 2015; Barrett et al. 2020; Brown et al. 2013) to understanding the infant feeding practices of Bangladeshi and Pakistani families living in the United Kingdom. This approach places heavy emphasis on not only the importance of community engagement but also the quality of it. Building trust with the women in the communities was crucial in order for them to feel like partners in the research and so that they felt comfortable sharing their personal stories, beliefs and practices. This was especially important given the sensitive topics relating to infant feeding behaviour. Exploring this allows for a high level of understanding and an informed steer for intervention development. Members of the community were actively involved throughout the project, engaging fully in the community workshops and demonstrating a commitment to all women's voices being heard and represented. Furthermore, the participating women were enthusiastic and seemed genuinely interested in developing a toolkit that could help others. Engaging the community, in which research intends to intervene, and following an evidence‐based framework, provides original and high‐quality data about the determinants of the behaviour specific to the target audience. This is one of the core recommendations for developing complex behavioural interventions (Skivington et al. 2021). Furthermore, exploring community and cultural level determinants alongside individual factors provides a more comprehensive consideration of the complex factors affecting particular behaviours and practices and their outcomes and increases the likelihood of an effective intervention approach. Findings were used to co‐develop an infant feeding intervention for Pakistani and Bangladeshi communities. Such interventions, mapped explicitly to address the determinants of behaviour and related social and cultural barriers as highlighted in this research have the potential to bridge a current gap in culturally appropriate support in the United Kingdom.

## 5 Conclusion

The LIFT project highlights the importance of high‐quality community engagement and the use of co‐development frameworks, such as the REPLACE approach (Barrett et al. 2015; Barrett et al. 2020; Brown et al. 2013), in the early stages of intervention development. The LIFT project provides valuable insight into infant feeding practices of women within Bangladeshi and Pakistani communities in the United Kingdom. Further, it identifies the communities' perspective on which infant feeding practices should be a priority for intervention and which are most amenable to change. The findings provide a robust evidence base on which to draw in the development of a culturally appropriate intervention toolkit to optimise infant feeding within these communities.

## Author Contributions

N.B., K.C., K.B., and J.B. conceptualised the study. All performed the research. N.B., K.K., and M.S. analysed the data. K.K. wrote the paper. K.B. provided feedback on first drafts. All read, and commented on drafts and approved the final manuscript.

## Conflicts of Interest

The authors declare no conflicts of interest.

## Data Availability

The authors have nothing to report.

## References

[mcn70009-bib-0001] Barrett, H. , K. Brown , Y. Alhassan , and D. Beecham . 2015. The REPLACE Approach: Supporting Communities to End FGM in the EU. A Toolkit. Coventry University. http://www.replacefgm2.eu/toolkit/default.aspx?section=50.

[mcn70009-bib-0002] Barrett, H. R. , K. Brown , Y. Alhassan , and E. Leye . 2020. “Transforming Social Norms to End FGM in the EU: An Evaluation of the REPLACE Approach.” Reproductive Health 17: 40. 32183828 10.1186/s12978-020-0879-2 PMC7079414

[mcn70009-bib-0003] Brown, K. , D. Beecham , and H. Barrett . 2013. “The Applicability of Behaviour Change in Intervention Programmes Targeted at Ending Female Genital Mutilation in the EU: Integrating Social Cognitive and Community Level Approaches.” Obstetrics and Gynecology International 2013: 1–12. 10.1155/2013/324362. PMC3745976 23983698

[mcn70009-bib-0004] Choudhry, K. 2017. *The Good milk: A Mixed Methods Approach to Understanding the Infant Feeding Outcomes, Influences and Experiences of Indian, Pakistani and Bangladeshi Mothers Living in the United Kingdom* (Issue December).

[mcn70009-bib-0005] Choudhry, K. , and L. M. Wallace . 2012. “‘Breast Is Not Always Best’: South Asian Women's Experiences of Infant Feeding in the UK Within an Acculturation Framework.” Maternal & Child Nutrition 8, no. 1: 72–87. 10.1111/j.1740-8709.2010.00253.x. 22136221 PMC6860851

[mcn70009-bib-0006] Cook, E. J. , F. Powell , N. Ali , C. Penn‐Jones , B. Ochieng , and G. Randhawa . 2021. “Improving Support for Breastfeeding Mothers: A Qualitative Study on the Experiences of Breastfeeding Among Mothers Who Reside in a Deprived and Culturally Diverse Community.” International Journal for Equity in Health 20, no. 1: 92. 10.1186/s12939-021-01419-0. 33823848 PMC8025360

[mcn70009-bib-0007] Craig, P. , P. Dieppe , S. Macintyre , S. Michie , I. Nazareth , and M. Petticrew . 2008. “Developing and Evaluating Complex Interventions: The New Medical Research Council Guidance.” BMJ (Clinical Research Ed.) 337: a1655. 10.1136/bmj.a1655. PMC2769032 18824488

[mcn70009-bib-0008] Hassan, S. M. , C. Leavey , J. S. Rooney , and S. Puthussery . 2020. “A Qualitative Study of Healthcare Professionals' Experiences of Providing Maternity Care for Muslim Women in the UK.” BMC Pregnancy and Childbirth 20: 400. 10.1186/s12884-020-03096-3. 32650735 PMC7350705

[mcn70009-bib-0009] Ingram, J. , K. Cann , J. Peacock , and B. Potter . 2008. “Exploring the Barriers to Exclusive Breastfeeding in Black and Minority Ethnic Groups and Young Mothers in the UK.” Maternal & Child Nutrition 4, no. 3: 171–180. 10.1111/j.1740-8709.2007.00129.x. 18582351 PMC6860569

[mcn70009-bib-0010] Ingram, J. , and D. Johnson . 2004. “A Feasibility Study of an Intervention to Enhance Family Support for Breast Feeding in a Deprived Area in Bristol, UK.” Midwifery 20, no. 4: 367–379. 10.1016/j.midw.2004.04.003. 15571885

[mcn70009-bib-0011] Ingram, J. , D. Johnson , and N. Hamid . 2003. “South Asian Grandmothers' Influence on Breast Feeding in Bristol.” Midwifery 19: 318–327. 10.1016/S0266-6138(03)00045-7. 14623511

[mcn70009-bib-0027] Kwah, K. , N. Bartle , M. Sharps , K. Choudhry , J. Blissett , and K. Brown . Forthcoming. Application of the Behaviour Change Wheel to Optimise Infant Feeding in Bangladeshi and Pakistani Communities in the UK: Co‐Development of the Learning About Infant Feeding Together (LIFT) intervention. 10.1111/mcn.70019 PMC12150127 40275628

[mcn70009-bib-0012] Lakhanpaul, M. , L. Benton , O. Lloyd‐Houldey , et al. 2020. “Nurture Early for Optimal Nutrition (NEON) Programme: Qualitative Study of Drivers of Infant Feeding and Care Practices in a British‐Bangladeshi Population.” BMJ Open 10, no. 6: e035347. 10.1136/bmjopen-2019-035347. PMC7307527 32565459

[mcn70009-bib-0013] Manikam, L. , R. Lingam , I. Lever , et al. 2018. “Complementary Feeding Practices for South Asian Young Children Living in High‐Income Countries: A Systematic Review.” Nutrients 10, no. 11: 1676. 10.3390/nu10111676. 30400582 PMC6266308

[mcn70009-bib-0014] Manikam, L. , A. Prasad , A. Dharmaratnam , et al. 2018. “Systematic Review of Infant and Young Child Complementary Feeding Practices in South Asian Families: The India Perspective.” Public Health Nutrition 21, no. 4: 637–654. 10.1017/S136898001700297X. 29166956 PMC10260904

[mcn70009-bib-0015] McAndrew, F. , J. Thompson , L. Fellows , A. Large , M. Speed , and M. J. Renfrew . 2012. *Infant Feeding Survey 2010*.

[mcn70009-bib-0016] McFadden, A. , M. J. Renfrew , and K. Atkin . 2013. “Does Cultural Context Make a Difference to Women's Experiences of Maternity Care? A Qualitative Study Comparing the Perspectives of Breast‐Feeding Women of Bangladeshi Origin and Health Practitioners.” Health Expectations 16, no. 4: e124–e135. 10.1111/j.1369-7625.2012.00770.x. 22429489 PMC5060684

[mcn70009-bib-0017] National Guideline Alliance [UK] . 2021. Breastfeeding Facilitators and Barriers: Postnatal Care: Evidence Review Q. National Institute for Health and Care Excellence (NICE). http://www.ncbi.nlm.nih.gov/books/NBK571561/. 34191455

[mcn70009-bib-0018] Office for Health Improvement and Disparities . 2021. Breastfeeding at 6 to 8 Weeks After Birth: Annual Data 2020 to 2021. GOV.UK. https://www.gov.uk/government/statistics/breastfeeding-at-6-to-8-weeks-after-birth-annual-data-2020-to-2021.

[mcn70009-bib-0019] Office for National Statistics . 2021. *2021 Census Area Profile—Coventry Local Authority*. https://www.nomisweb.co.uk/sources/census_2021/report?compare=E08000026#section_5.

[mcn70009-bib-0020] Plested, B. A. , R. W. Edwards , and P. Jumper‐Thurman . 2006. *A Handbook for Successful Change*.

[mcn70009-bib-0021] Rayment, J. , C. McCourt , L. Vaughan , J. Christie , and E. Trenchard‐Mabere . 2016. “Bangladeshi Women's Experiences of Infant Feeding in the London Borough of Tower Hamlets.” Maternal & Child Nutrition 12, no. 3: 484–499. 10.1111/mcn.12169. 25684682 PMC6860151

[mcn70009-bib-0022] Skivington, K. , L. Matthews , S. A. Simpson , et al. 2021. “A New Framework for Developing and Evaluating Complex Interventions: Update of Medical Research Council Guidance.” BMJ (Clinical Research ed.) 374: n2061. 10.1136/bmj.n2061. PMC8482308 34593508

[mcn70009-bib-0023] Twamley, K. , S. Puthussery , S. Harding , M. Baron , and A. Macfarlane . 2011. “UK‐Born Ethnic Minority Women and Their Experiences of Feeding Their Newborn Infant.” Midwifery 27, no. 5: 595–602. 10.1016/j.midw.2010.06.016. 21035928

[mcn70009-bib-0024] Victora, C. G. , R. Bahl , A. J. D. Barros , et al. 2016. “Breastfeeding in the 21st Century: Epidemiology, Mechanisms, and Lifelong Effect.” Lancet 387, no. 10017: 475–490. 10.1016/S0140-6736(15)01024-7. 26869575

[mcn70009-bib-0025] Wilde, M. 2023. “Culture: A Determinant of Breastfeeding in SCPHN Practice.” British Journal of Child Health 4, no. 6: 277–281. 10.12968/chhe.2023.4.6.277.

[mcn70009-bib-0026] World Health Organisation . 2003. *Global Strategy for Infant and Young Child Feeding: Report by the Secretariat. 1*, 5.

